# Total brachial artery reactivity and first time incident coronary heart disease events in a longitudinal cohort study: The multi-ethnic study of atherosclerosis

**DOI:** 10.1371/journal.pone.0211726

**Published:** 2019-04-10

**Authors:** Joseph F. Polak, Pamela Ouyang, Dhananjay Vaidya

**Affiliations:** 1 Ultrasound Reading Center, Department of Radiology, Tufts Medical Center, Boston, MA, United States of America; 2 Department of Medicine, Johns Hopkins University School of Medicine, Baltimore, MD, United States of America; University of Perugia, ITALY

## Abstract

**Background:**

Brachial artery reactivity (BAR) is usually determined as the maximum brachial artery diameter (BAD) following release of an occluding pressure cuff compared to a BAD before cuff inflation. BAD early after cuff deflation can also serve as baseline for estimating total brachial artery reactivity (TBAR). We investigate whether TBAR is associated with first time coronary heart disease events.

**Methods:**

Participants of the Multi-Ethnic Study of Atherosclerosis (n = 5499) consisting of whites, African-Americans, Chinese and Hispanics were followed longitudinally for a mean of 12.5 years. Brachial artery ultrasound was performed following five minutes of cuff occlusion at the forearm. TBAR was estimated from BAD following cuff release as the difference between maximum and minimum brachial artery diameters divided by the minimum diameter multiplied by 100%. TBAR was added to multivariable Cox proportional hazards models with Framingham risk factors as predictors and time to first coronary heart disease event as outcome.

**Results:**

Average TBAR was 9.7% (9.7 SD). Mean age was 61.7 years, 50.9% women. Increased TBAR was associated with lower risk of CHD events with a hazard rate of 0.78 per SD increase (95% C.I. 0.67, 0.91; p = 0.001). A TBAR below the median of 7.87% (Inter Quartile Range: 4.16%, 13.0%) was associated with a 31% lower risk of coronary heart disease event (Hazard Ratio: 0.69; 95% C.I.: 0.55, 0.87).

**Conclusion:**

TBAR is an independent predictor of first time coronary heart disease events and is exclusively measured after release of a blood pressure occlusion cuff.

## Introduction

The brachial artery dilates in response to the endogenous release of nitric oxide (NO) that occurs during reactive hyperemia [1]. Brachial artery flow-mediated dilation (FMD), also called brachial artery reactivity (BAR), is typically seen on ultrasound imaging following the release of an occlusive blood pressure cuff that has been kept inflated for five minutes in order to induce forearm ischemia. An increase in brachial artery diameter is a marker of an “healthy endothelium” while lessened degrees of diameter increase are associated with increased risk of cardiovascular outcomes [2]. The calculation of BAR typically relies on obtaining a baseline diameter before cuff inflation [3] and is ideally done with the aid of a stereotactic device that stabilizes the location of the ultrasound imaging probe over the brachial artery [1, 4].

While most investigators use baseline brachial artery diameters before cuff inflation for BAR calculations, an alternate approach is to use a baseline brachial artery diameter following cuff deflation [5–8]. This approach can help compensate for the absence of a stereotactic device and lessen the possibility of inadvertent probe displacement while the occlusion cuff is inflated or when it is deflated.

The brachial artery responds to vasodilator stimuli in a fashion similar to the coronary artery [9]. As such, it is considered a surrogate for the effects of atherosclerosis on the coronary artery. This hypothesis is supported by outcomes studies linking traditional FMD measurements with coronary heart disease events [10].”

We propose to measure total brachial artery reactivity (TBAR) as the difference between the minimum and maximum brachial artery diameters following release of the occlusion cuff divided by the minimum diameter. No paper has investigated whether this response is associated with incident coronary heart disease (CHD) events in individuals free of CHD.

We hypothesize that TBAR is independently associated with first time coronary heart disease events in the Multi-Ethnic Study of Atherosclerosis (MESA), a longitudinal follow-up study of a multi-ethnic cohort of individuals free of CHD at baseline.

## Materials and methods

### Population

MESA is a multiethnic population of 6814 men and women aged 45–84 with no history of clinical cardiovascular disease [11] recruited between July 2000 and August 2002. MESA is a cohort that includes white, African-American, Hispanic-American, and Chinese participants from six separate sites in the United States. Participants were excluded from enrollment if they had a physician diagnosis of heart attack, stroke, transient ischemic attack, heart failure, angina, atrial fibrillation or a history of any cardiovascular procedure, a weight above 300 lbs, pregnancy, or any medical conditions that would prevent long-term participation. MESA protocols and all studies described herein have been approved by the Institutional Review Boards of all collaborating institutions in the United States: Columbia University, New York NY; Johns Hopkins University, Baltimore MD; Northwestern University, Chicago IL; University of California at Los Angeles CA; University on Minnesota, Twin Cities MN; and Wake Forest University, Winston-Salem NC. The participants gave informed consent and underwent an evaluation of brachial artery endothelial function at the baseline visit.

**Fig 1:**
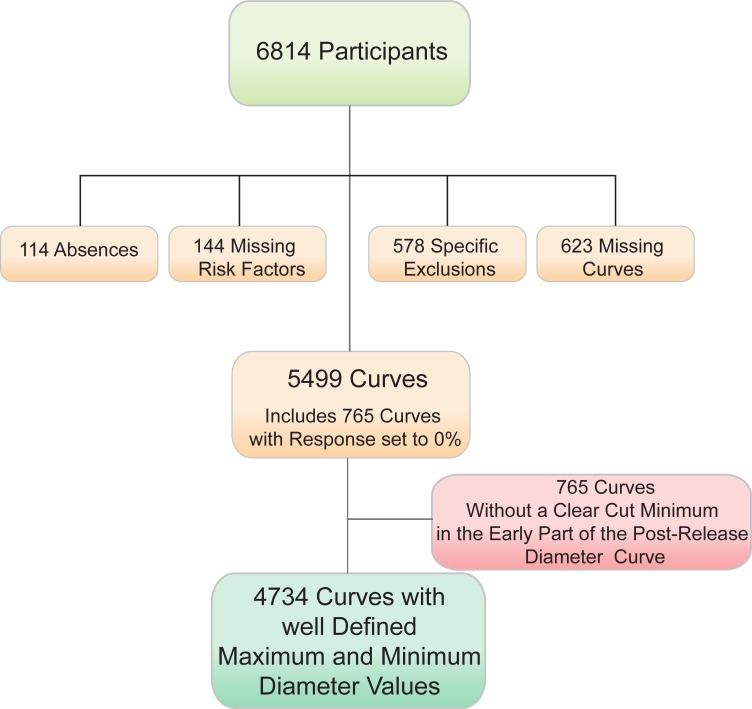
Study cohort composition. Various physiological and technical issues lead to the exclusion of 578 individuals as listed in the text. In addition, 144 participants had incomplete risk factor profiles, 114 failed to show up at the examination site. Digital copies of brachial artery diameter curves were not available in 623 cases. As discussed later, 5499 processed curves were analyzed. Of these, 4734 had the expected time course of an increase in diameter at least 30 seconds following cuff release preceded by a minimum diameter. This pattern was not seen on the remaining 765 curves where a maximum diameter was not seen starting 20 seconds after cuff release.

The population studied was further restricted to individuals with available brachial artery diameter tracings, complete Framingham risk factor evaluations (n = 5499; Fig 1). The 578 cases with specific exclusions included 140 individuals with systolic blood pressures of 180 mmHg or above, a blood pressure difference between both arms > 15 mmHg (n = 151), 97 with prior mastectomy, various medical issues with the right hand or arm (n = 31), Raynaud’s phenomenon in 54 individuals, patient related factors (n = 64) and 41 instances of technical difficulties. The identification of minimum and maximum diameters within defined time windows following the release of an occlusion cuff was possible in 4734 individuals (Fig 1).

### Risk factors and anthropomorphic variables

The risk factors used in this paper are derived from the original CHD Framingham Risk Score [12]: age, smoking and diabetes status, systolic blood pressure, LDL and HDL cholesterol with sex and race/ethnicity added.

Age, sex, race/ethnicity, and medical history were self-reported. Current smoking was defined as self-report of a cigarette in the last 30 days. Resting blood pressure (BP) was measured in the seated position using a Dinamap model Pro 100 automated oscillometric sphygmomanometer (Critikon, Tampa, Florida); pressures were the average of the last two of three performed measurements. Lipid levels were measured after a twelve-hour fast. Total cholesterol was measured using a cholesterol oxidase method (Roche Diagnostics), as was HDL cholesterol following precipitation of non HDL-cholesterol with magnesium/dextran. Triglycerides were measured with Triglyceride GB reagent (Roche Diagnostics) and LDL cholesterol estimated [13]. The presence of diabetes mellitus was based on self-reported physician diagnosis, use of insulin and/or oral hypoglycemic agent, or a fasting glucose value ≥126 mg/dL [14].

### Total brachial artery reactivity measurements

Trained technicians at each of the six field centers acquired B-mode ultrasound images with a Logiq-700 ultrasound device (GE Healthcare, Waukesha, WI) and an ultrasound transducer (M12L) set at 9 MHz. The sonographers performed the study by holding the transducer and did not have access to a stereotactic holder. The ultrasound probe was placed on the medial aspect of the right arm a few centimeters above the elbow with a slight angulation in order to best visualize the brachial artery. An occlusion cuff was placed on the upper right forearm. A blood pressure cuff was inflated to a pressure 50 mm Hg above maximal systolic pressure. The cuff was kept inflated for 5 minutes with the ultrasound probe held centered over the same brachial artery segment. Images were videotaped starting 15 seconds before cuff deflation and continuing for 90 seconds after cuff release. Videotape recordings were made using super VHS tapes.

**Fig 2:**
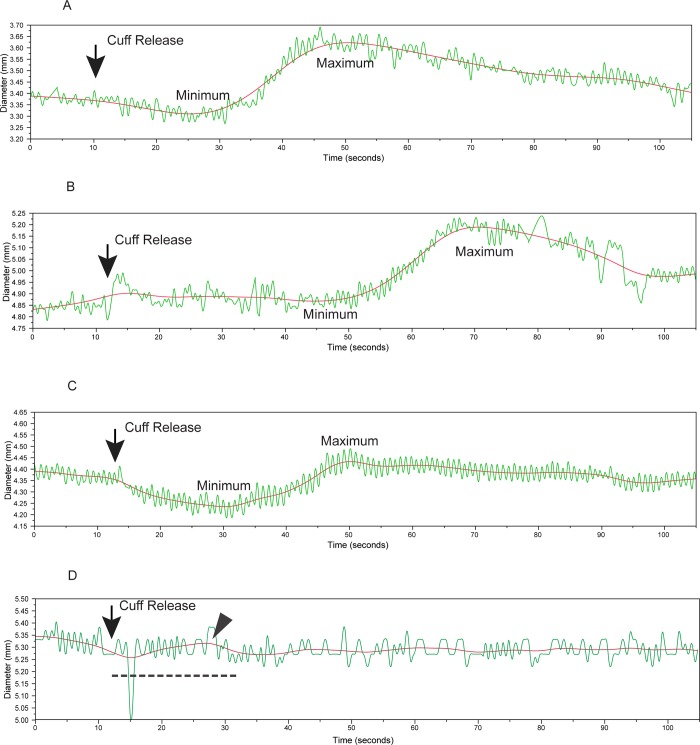
Total brachial artery reactivity (TBAR) after the release of an occlusion cuff inflated for 5 minutes. The rapidly varying diameter values are raw diameter measurements (green) while the solid smooth lines (red) are the results of curve smoothing. Maximum and minimal brachial artery diameters were made from the smoothed curves. TBAR is the difference between maximum and minimum diameters divided by the minimum diameter and then multiplied by 100%. Visual review of some of the studies showed subjective differences in the responses: (A) a strong decrease in brachial artery diameter followed by a strong maximum; (B) a small decrease in diameter followed by a strong increase; and (C) a marked decrease in diameter followed by a small increase. Equivocal results (D) in 765 instances (765/5499: 13.9%) were due to a failure to detect a minimum diameter in the 20 seconds following cuff release (dotted line) since at least one diameter value within this time window (arrowhead) was greater than subsequent diameters.

The acquired images were sent to Tufts Medical Center Ultrasound Reading Center for blinded processing. Digital streams of the brachial artery ultrasound images were acquired from the videotapes at a frame-rate of 30 frames-per-second as MJPEG compressed images (compression ratio six to one) using a Pinnacle DC-30 Video board (Corel Inc., Mountain View CA) and a Compaq AP-200 workstation (Compaq Computer Corporation, Houston, TX) equipped with a Pentium III processor (Intel Corporation, Santa Clara, CA). A reader reviewed the images and identified the point at which the blood pressure cuff had been released. The reader then identified an appropriate brachial artery segment and placed a rectangular region-of-interest on a selected image frame. Customized software was used to calculate the location of the near and far wall media-adventitia interfaces in this region-of-interest and to generate brachial artery diameter versus time curves without manual editing. These were transferred to Access (Microsoft, Redmond WA) databases for archiving. The archived brachial artery diameter curves were subsequently retrieved and processed using a MATLAB (The MathWorks Inc., Natick MA) program that smoothed the diameter versus time curves using a finite impulse response digital filter and processed the resultant curves to identify the maximum diameter and time to maximum diameter starting 20 seconds following release of the blood pressure cuff. The algorithm then searched for a minimum diameter going backwards until 10 seconds after cuff release (Fig 2A–2C) based on previous observations [5, 6]. Responses were reported as (maximum diameter–minimum diameter) / (minimum diameter) multiplied by 100% or total brachial artery reactivity (TBAR). Although we processed 5499 brachial diameter curves, there were 765 instances where the curve analysis algorithm detected a maximum diameter in a time window 20 to 30 seconds after cuff release and failed to find a smaller diameter in the preceding time interval (Fig 2D). These cases likely represented low amplitude responses. We therefore assigned them a zero TBAR value and included them in the primary analyses.

Reproducibility was assessed by blinded review of replicate studies performed on the same participant and acquired on the same day (n = 88). Because of the blinded design, the same reader performed the diameter extractions on participants with “dummy” identification numbers. The correlation coefficient for determining the maximum brachial artery diameter was 0.90 (95% CI: 0.85, 0.94). Replicate measurements of TBAR had a moderate correlation coefficient of 0.50 (95% CI: 0.33, 0.64). The mean TBAR at the first acquisition was 11.2% and 10.9% for the second acquisition for a non-significant difference of 0.2% (95% CI: -1.2%, 0.7%).

### Software validation

Brachial artery baseline diameters and then peak-diameters following release of the occlusion cuff were measured using three approaches: 1) the current software, named Funky Work Station (FWS), was used to extract the diameter curves that were subsequently processed to generate TBAR, 2) Brachial Analyzer, a commercial widely distributed software tool (Vascular Research Tools 5; Medical Imaging Application, LLC, Coralville, IA, USA) and, 3) the software used to measure FMD in the Multi-Ethnic Study of Atherosclerosis (MESA) and the Cardiovascular Health Study (CHS)[15–17]. Baseline (BASE) BAD measurements and peak-response (PR) BAD were used to calculate FMD according to the equation: FMD = ((PR BAD–BASE BAD)/ BASE BAD)) *100.

Studies performed on 90 participants were used. Mean age was 61.8 years (10.8 SD). Fifty-six percent were women and the race/ethnicity breakdown was 40% non-Hispanic whites, 11.1% Chinese-American, 30% African-American and 18.9% Hispanic American. The same operator used the FWS software and Brachial Analyzer, while one different reader used the MESA FMD software. In all three instances, the videotaped image sequences were digitized and then processed by the respective software tools.

To further test the predictive value of TBAR against the more traditional method of estimating FMD, we obtained the original MESA FMD dataset, hereon referred to as “classic” FMD. Of the 3501 entries in this dataset, there were 3162 individuals also having TBAR measurements.

### Coronary heart disease events

Events were identified during follow-up examinations and by telephone interview conducted every 9 to 12 months to inquire about all interim hospital admissions, cardiovascular outpatient diagnoses, and deaths. Copies were obtained of all death certificates and of all medical records for hospitalizations and outpatient cardiovascular diagnoses. Two physicians from the MESA study events committee independently reviewed all medical records for end-point classification and assignment of incidence dates. Coronary heart disease (CHD) events included myocardial infarction, resuscitated cardiac arrest, and death secondary to coronary heart disease. A total of 16 suspected CHD events occurred in close proximity to the participant enrollment date. These participants were excluded from the analyses.

### Statistical analyses

Variables are presented as means and standard deviation (SD) values if continuous and as percentages if categorical. Median values are shown with inter-quartile ranges (IQR).

The baseline multivariable Cox proportional hazards regression model used robust error handling to take account of outliers. The model was created with the components of the traditional Framingham risk score for coronary artery disease: age, systolic blood pressure, diabetes, HDL-cholesterol, LDL-cholesterol and smoking history. We added sex and race/ethnicity.

TBAR measurements were added to the baseline model and the hazards ratio for TBAR obtained. We reported the results for continuous variables by their respective standard deviation values. Kaplan-Meier survival rates were plotted by quartiles of TBAR values.

We assigned a TBAR value of 0.0% to the participants with low amplitude responses (Fig 2D) for a full analytical sample of 5499 participants and then excluded 16 cases with outcomes near enrolment. We also performed analyses in the subset of 4734 participants (excluding 15 participants with early outcomes for a total of 4719) whose curves showed a clear minimum and maximum diameter response.

We looked at possible outliers by setting two plausible boundary values i.e., at 0% if below zero and 40.93% if above the 99^th^ percentile. We repeated the analysis after excluding the outliers.

We separately compared the measurements made with FWS, the current software, with Brachial Tools and the MESA FMD software. We also compared the results of Brachial Analyzer with those of the MESA FMD software. In all three instances, the intra-class correlation coefficient (ICC) was calculated and the 95% confidence intervals generated using a mixed model, i.e., the software tool was considered a “fixed” effect. Since the purpose was to compare to overall reproducibility of obtaining brachial artery diameters, we reported the ICC for the mean response. We further set to zero all negative responses seen with FWS and Brachial Analyzer since this was the convention used for MESA FMD.

Finally, we compared “traditional” FMD to TBAR with two Cox proportional hazards models. In the first we used TBAR (per 1 SD value of 10.5%) as predictor and time to CHD event as outcome. In the second model we used “classic” FMD (per 1 standard deviation value of 8.7%) as the predictor. In both cases we report the hazard ratios, calculated the C-statistics and compared them. We repeated these analyses in minimally adjusted models: age, sex, and race-ethnicity.”

Analyses were performed using Stata version 11.2 (Stata Corporation, College Station, TX). Level of significance was two-sided at p < 0.05.

## Results

### Total brachial artery reactivity

Average participant age was 62.0 years (10.2 years SD) with 50.9% women (Table 1). The mean cohort follow-up was 12.5 years. The ethnic composition of the cohort was white (36.4%), Chinese, (13.4%) African-American (27.8%) and Hispanic (22.4%). The distribution of Framingham risk factors is shown in Table 1. Mean TBAR was 9.7% (± 9.7% SD). The maximal brachial artery diameter was 4.80 mm. Because of missing minimal diameter values, the other brachial artery reactivity parameters will be shown in Table 2. A total of 328 first time coronary artery disease events occurred during follow-up.

**Table 1:** Demographics and Framingham risk factors for all participants with brachial artery diameter curves.

Variable	Values (n = 5483)†
Age (years)	62.0 (10.2 SD)
Race/ Ethnicity	
White	1,995 (36.4%)
Chinese	733 (13.4%)
African-American	1524 (27.8%)
Hispanic	1231 (22.4%)
Sex (women)	2789 (50.9%)
Smoker	
Never	2809 (51.1%)
Prior	2003 (36.4%)
Current	685 (12.5%)
Diabetes (yes)	518 (9.5%)
Systolic Blood Pressure (mmHg)	125.4 (20.0 SD)
LDL cholesterol (mg/dL)	117.3 (31.4 SD)
HDL cholesterol (mg/dL)	50.8 (14.6 SD)
Maximal brachial artery diameter (mm)	4.80 (0.88 SD)
Total Brachial Artery Reactivity (%)	Mean: 9.7 (9.7 SD)
	Median: 7.87 (IQR: 4.16, 13.0)
Coronary Heart Disease Events	328 (6.0%)
Follow-up (years)	Mean: 12.5 (3.6 SD); median: 14.0 (IQR: 12.4, 14.7)

† Out of 5499 participants, there were 16 instances of early events near enrollment. These cases were excluded in subsequent analyses.

SD corresponds to standard deviation while IQR indicates inter-quartile range.

**Table 2:** Demographics and Framingham risk factors for participants included in the study who had distinct measurable minimal and maximal diameters.

Variable	Values (n = 4719)†
Age (years)	61.7 (10.3 SD)
Race/ Ethnicity	
White	1,775 (37.6%)
Chinese	620 (13.1%)
African-American	1243 (26.4%)
Hispanic	1081 (22.9%)
Sex (women)	2389 (50.6%)
Smoker	
Never	2429 (51.2%)
Previous	1721 (36.4%)
Current	588 (12.4%)
Diabetes (yes)	432 (9.2%)
Systolic Blood Pressure (mmHg)	124.7 (19.7 SD)
LDL cholesterol (mg/dL)	117.5 (31.4 SD)
HDL cholesterol (mg/dL)	50.6 (14.5 SD)
Maximum brachial artery diameter (mm)	4.90 (0.87 SD)
Minimum brachial artery diameter (mm)	4.33 (0.86 SD)
Time to Maximum Diameter (sec)	55.2 (14.6 SD)
Time to Minimum Diameter (sec)	19.4 (8.9 SD)
Total Brachial Artery Reactivity (%)	Mean: 11.3 (9.6 SD)
Total Brachial Artery Reactivity (%)	Median: 9.03 (IQR: 5.78, 14.1)
Coronary Heart Disease Events	275 (5.8%)
Follow-up (years)	Mean: 12.6 (3.5 SD); median: 14.1 (IQR: 12.7, 14.7)

† Out of 4734 participants, there were 15 instances of events occurring near enrollment. These cases were excluded.

SD corresponds to standard deviation while IQR indicates inter-quartile range.

The distribution of risk factors for the 4719 participants who had clearly identified maximal and minimal diameters following release of the occlusion cuff is shown in Table 2. Overall patient demographics were similar to those of the full cohort (Table 1).

Average maximum diameter at peak-artery dilation was 4.90 mm (0.87 mm SD) and the early minimum diameter was 4.33 mm (0.86 mm SD). Average time taken to attain maximum artery diameter was 55.2 (±14.6 SD) seconds and 19.4 sec ± 8.9 SD) seconds for minimum diameter. Mean TBAR was 11.3% (± 9.6% SD). The number of events decreased to 275 for a similar follow-up interval.

The results of the multivariable model are shown in Table 3. All traditional Framingham risk factors were significantly associated with incident coronary heart disease with the exception of LDL cholesterol. TBAR was a significant independent predictor of lower risk of CHD when added to the Framingham risk factors with a hazard ratio of 0.78 for each 9.6% increase (p = 0.003). This corresponds to a slightly larger than 2% decrease in risk for each percent increase in TBAR.

**Table 3:** This table shows the results of a multivariable Cox proportional model including total brachial artery reactivity (TBAR) and traditional Framingham risk factors for all participants with complete data as shown in Table 2.

Variable	Hazard Ratio	Lower 95% CI	Upper 95% CI	p-value
Age (years)	1.06	1.05	1.08	< 0.001
Sex (male)	1.64	1.27	2.11	< 0.001
Current Smoker (yes)	1.71	1.25	2.33	0.001
Diabetes (yes)	1.70	1.24	2.32	0.001
Systolic Pressure (20.0 mmHg)†	1.25	1.11	1.40	< 0.001
LDL cholesterol (31.4 mg/dL)†	1.05	0.94	1.19	0.38
HDL cholesterol (14.6 mg/dL)†	0.80	0.70	0.92	0.002
Total Brachial Artery Reactivity (%)†	0.78	0.67	0.91	0.001

Population size is 5483 (5499 discounting 16 events close to enrollment).

† Reported for a change of one standard deviation.

765 participants had low amplitude TBAR responses. These TBAR values were assigned a value of 0.0%. The statistical model is also adjusted for race/ethnicity.

Similar results were obtained when we repeated the analyses using the median TBAR as a marker (Table 4). The risk of CHD events in the full cohort decreased 31% when participants with a TBAR greater than 7.87% were compared to those with values below the median.

**Table 4:** This table shows the effect of adding the median of total brachial artery reactivity to a model with Framingham risk factors and the full participant population shown in Tables 1 and 3.

Variable	Hazard Ratio	Lower 95% CI	Upper 95% CI	p-value
Age (years)	1.06	1.05	1.08	< 0.001
Sex (male)	1.65	1.28	2.13	< 0.001
Current Smoker (yes)	1.73	1.27	2.36	0.001
Diabetes (yes)	1.70	1.24	2.33	0.001
Systolic Pressure (20.0 mmHg)†	1.25	1.11	1.40	< 0.001
LDL cholesterol (31.4 mg/dL)†	1.06	0.94	1.19	0.36
HDL cholesterol (14.6 mg/dL)†	0.81	0.70	0.93	0.002
Median total brachial artery reactivity (%)	0.69	0.55	0.87	0.001

Population size is 5483 (5499 discounting 16 events close to enrollment).

† Reported for a change of one standard deviation.

765 participants had low amplitude TBAR responses possibly due to noise or low amplitude responses; the TBAR values were assigned a value of 0.0%.

Statistical model is also adjusted for race/ethnicity.

We repeated the analysis restricting the study population to the participants with clearly defined minima and maxima on their brachial artery diameter curves (Table 5). Again, all traditional Framingham risk factors were significantly associated with incident coronary heart disease with the exception of LDL cholesterol. TBAR remained a significant independent predictor of lower risk of CHD when added to the Framingham risk factors with a hazard ratio of 0.73 for each 9.6% increase (p = 0.003). This corresponds approximately to a 3% decrease in risk for each percent increase in TBAR.

**Table 5:** Results of multivariable Cox proportional hazards models predicting first coronary artery disease event with Total Brachial Artery Reactivity (TBAR) in a model with traditional Framingham risk factors in the participants with brachial artery diameter curves with clear minima and maxima.

Variable	Hazard Ratio	Lower 95% CI	Upper 95% CI	p-value
Age (years)	1.06	1.05	1.08	< 0.001
Sex (male)	1.60	1.21	2.11	0.001
Current Smoker (yes)	1.98	1.42	2.76	< 0.001
Diabetes (yes)	1.82	1.28	2.57	0.001
Systolic Pressure (19.7 mmHg)†	1.28	1.13	1.45	< 0.001
LDL-cholesterol (31.4 mg/dL)†	1.04	0.91	1.18	0.58
HDL-cholesterol (14.5 mg/dL)†	0.78	0.67	0.91	0.002
Total Brachial Artery Reactivity (9.6%)†	0.73	0.61	0.89	0.001

Population size is 4719. Statistical model is also adjusted for race/ethnicity.

† Reported for a change of one standard deviation.

Repeating the analysis with median TBAR as a cut-point showed that total brachial artery reactivity remained significantly associated with events, hazard ratio of 0.66 (95% CI: 0.51, 0.85; p = 0.001), when participants with a TBAR above the median were compared to those below (Table 6). This corresponds to a decreased risk of 34% for individuals with a TBAR above the median of 9.04%.

**Table 6:** Results of a multivariable Cox proportional hazards model predicting first coronary artery disease event stratifying Total Brachial Artery Reactivity (TBAR) above and below the sample median in a model with traditional Framingham risk factors.

Variable	Hazard Ratio	Lower 95% CI	Upper 95% CI	p-value
Age (years)	1.06	1.05	1.08	< 0.001
Sex (male)	1.60	1.21	2.13	0.001
Current Smoker (yes)	1.99	1.43	2.77	< 0.001
Diabetes (yes)	1.80	1.27	2.55	0.001
Systolic Pressure (19.7 mmHg)†	1.28	1.13	1.44	< 0.001
LDL cholesterol (31.4 mg/dL)†	1.04	0.91	1.18	0.57
HDL cholesterol (14.5 mg/dL)†	0.78	0.67	0.91	0.002
Total Brachial Reactivity (TBAR) above versus below the median)	0.66	0.51	0.85	0.001

Population size is 4719. Statistical model is adjusted for race/ethnicity.

† Reported for a change of one standard deviation.

The median value of Total Brachial Artery Reactivity is 9.04% (IQR: 5.8, 14.1).

**Fig 3:**
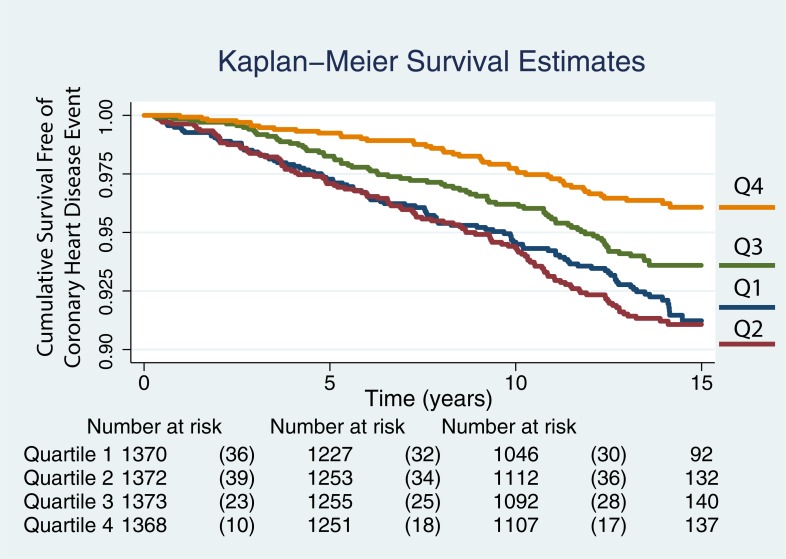
Kaplan-Meier curves showing the likelihood of remaining event free with time. The curves for the event free survival of all individuals are plotted by quartiles, quartile 1 (Q1) being the lowest and Q4 the largest. The plotted quartiles were -5.18 to 4.16%, 4.16 to 7.78%, 7.87 to 13.02%, and 13.02 to 190.22%.

The Kaplan-Meier curves for TBAR quartiles are shown in Fig 3 for all 5483 participants. There is decreased risk of a CHD event with time in individuals with the highest two quartiles while the two lowest quartiles show poor discrimination. The plotted quartiles were -5.18 to 4.16%, 4.16 to 7.78%, 7.87 to 13.02%, and 13.02 to 190.22%

### Outlier analyses

A sensitivity analysis was performed accounting for plausible outliers. The one negative TBAR value and the 59 values above the 99^th^ percentile (40.93%) were excluded. The Cox proportional hazards model was then applied to the data (Table 7). The mean TBAR was now 9.46% (8.0% SD). The TBAR hazard ratio increased to 0.81, i.e. a decrease risk of 19% for a TBAR but remained statistically significant at p = 0.002.

**Table 7:** Results of multivariable Cox proportional hazards models predicting first coronary artery disease event with Total Brachial Artery Reactivity (TBAR) in a model with traditional Framingham risk factors in all participants after accounting for outliers.

Variable	Hazard Ratio	Lower 95% CI	Upper 95% CI	p-value
Age (years)	1.06	1.05	1.08	< 0.001
Sex (male)	1.64	1.28	2.09	< 0.001
Current Smoker (yes)	1.71	1.25	2.34	0.001
Diabetes (yes)	1.70	1.26	2.30	0.001
Systolic Pressure (20.0 mmHg)†	1.25	1.12	1.40	< 0.001
LDL-cholesterol (31.4 mg/dL)†	1.05	0.94	1.18	0.35
HDL-cholesterol (14.6 mg/dL)†	0.80	0.70	0.92	0.001
Total Brachial Artery Reactivity (8.0%)†	0.81	0.71	0.92	0.002

Population size is 5423. Statistical model is also adjusted for race/ethnicity.

† Reported for a change of one standard deviation.

**Fig 4:**
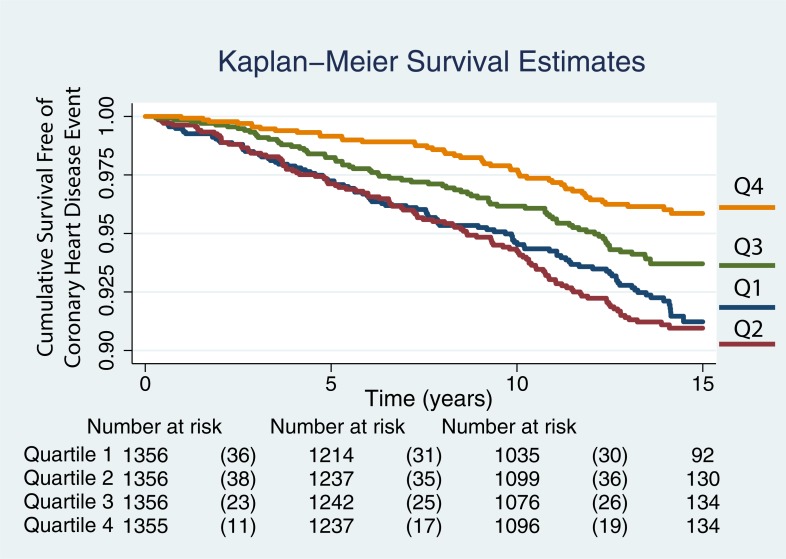
Kaplan-Meier curves showing the likelihood of remaining event free with time for TBAR values after excluding 60 outliers. The curves for the event free survival of 5423 individuals after excluding outliers are plotted by quartiles, quartile 1 (Q1) being the lowest and Q4 the largest. The TBAR quartiles were 0.0 to 4.13%, 4.13 to 7.79%, 7.79 to 12.81%, and 12.81 to 40.11%.

The Kaplan-Meier curves for TBAR quartiles are shown in Fig 4 for the 5423 participants after excluding outliers. The hazard ratio was 0.77 (95% CI: 0.66, 0.90) for TBAR after excluding all 60 outliers and remained significant at p = 0.0001 for a mean TBAR of 9.15% (7.38 SD). As before, there is decreased risk of a CHD event with time in individuals with the highest two quartiles while the two lowest quartiles show poor discrimination. The TBAR quartiles were 0.0 to 4.13%, 4.13 to 7.79%, 7.79 to 12.81%, and 12.81 to 40.11%.

**Fig 5:**
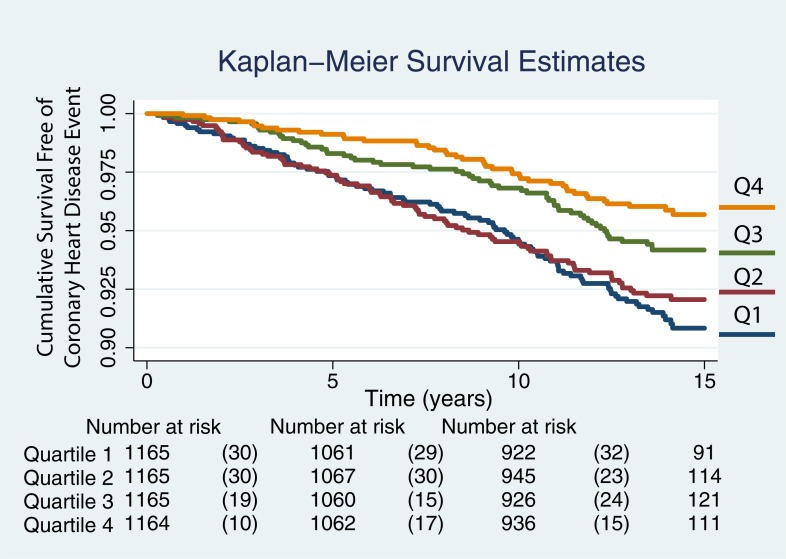
Kaplan-Meier curves showing the likelihood of remaining event free with time for TBAR values after excluding 60 outliers, instances of poorly defined maxima and minima, and early events. The curves for the event free survival of 4659 individuals after excluding outliers are plotted by quartiles, quartile 1 (Q1) being the lowest and Q4 the largest. The TBAR quartiles were 0.0 to 5.75%, 5.76 to 8.95%, 8.95 to 13.9%, and 13.9 to 40.11%.

The Kaplan-Meier curves for TBAR quartiles are shown in Fig 5 for the 4659 participants after excluding outliers, instances of low amplitude responses and events noted at enrollment. As before, there is decreased risk of a CHD event with time in individuals with the highest two quartiles while the two lowest quartiles now show better scaling for events than on Fig 4. The TBAR quartiles were 0.0 to 5.75%, 5.76 to 8.95%, 8.95 to 13.9%, and 13.9 to 40.11%.

### Software validation

The base, post-release brachial artery diameters as measured by the three different software tools, FWS, Brachial Analyzer, and MESA FMD and the calculated FMD values are shown in Table 8. The brachial artery diameters are larger and the calculated FMD is smaller for FWS when compared to the two other tools.

**Table 8:** Mean brachial artery diameter (BAD) and flow mediated dilation (FMD) and standard deviation values for three software tools: Brachial Analyzer (BA), the one used in this study, Funky Work Station (FWS), and the Multi-Ethnic Study of Atherosclerosis (MESA) FMD software tool.

Measurement	BA	FWS	MESA FMD
Base BAD (mm)	4.27 (±0.85)	4.42 (±0.84)	4.21 (±0.81)
Peak-Response BAD (mm)	4.46 (±0.83)	4.56 (±0.83)	4.40 (±0.78)
Flow-Mediated Dilation (%)	5.6 (± 5.9)	3.6 (± 3.3)	4.9 (±3.4)

The ICCs between FWS, Brachial Analyzer and MESA FMD are shown in Table 9. In summary, the ICCs for diameters were all greater than 0.9 for BASE BAD and PR BAD. However, while the estimated FMD between FWS FMD and MESA FMD was moderate at 0.76 (95% confidence intervals: 0.64, 0.84), the ICCs between these two tools and Brachial Analyzer were weaker.

**Table 9:** Intra-class correlations coefficients (ICC) and 95% confidence intervals (95% CI) between baseline (BASE) brachial artery diameter (BAD), peak response (PR) BAD, and estimated flow-mediated dilation (FMD) between Brachial Analyzer (BA), Funky Work Station (FWS), and the Multi-Ethnic Study of Atherosclerosis (MESA) FMD software tools.

Type of ICC	Base BAD (mm)	Peak-Response BAD (mm)	Flow-Mediated Dilation (%)
BA versus FWS (95% CI)	0.95 (0.92, 0.97)	0.93 (0.89, 0.95)	0.47 (0.20, 0.65)
BA versus MESA FMD (95% CI)	0.91 (0.86, 0.94)	0.90 (0.85, 0.93)	0.38 (0.06, 0.59)
FWS versus MESA FMD (95% CI)	0.94 (0.92, 0.96)	0.94 (0.91, 0.96)	0.76 (0.64, 0.84)

Comparing TBAR to “classic” FMD in an un-adjusted model, TBAR had a hazard ratio of 0.73 (95% CI: 0.60, 0.90) for a p = 0.002, similar to that of FMD with a hazard ratio of 0.78 (95% CI: 0.87, 0.90), p = 0.001. The respective C-statistics were 0.566 (95% CI: 0.53, 0.602), p < 0.001 and 0.566 (95% CI: 0.529, 0.603). The differences between both were not significant (p = 0.99). In the minimally adjusted models, TBAR had a lower hazard ratio (HR 0.83; 95% CI: 0.69, 0.99) than FMD (HR 0.94; 95% CI: 0.81, 1.10). However TBAR was a borderline significant predictor (p = 0.049) of events whereas FMD was not (p = 0.44). The C-statistics for both models were similar and not statistically different (p = 0.35) with the TBAR model having 0.716 (95% CI: 0.683, 0.749) and the FMD model 0.713 (95% CI: 0.680, 0.746).

## Discussion

We have measured total brachial artery reactivity (TBAR) by relying on the brachial artery diameters obtained following the release of a blood pressure occlusion cuff. We have shown that this measurement is an independent predictor of future coronary heart disease events in a population free of cardiovascular disease at baseline after accounting for the Framingham risk factors.

Our measurement process is different from the method typically used in the assessment of flow mediated brachial artery dilation / brachial artery reactivity (FMD / BAR) since we obtained the baseline diameters used to calculate TBAR after cuff release and not before cuff inflation [1, 3]. TBAR as measured by us includes a previously described early decrease in artery diameter following release of the occlusion cuff [5–8]. This diameter decrease is believed to represent a form of brachial artery vasoconstriction [6, 7]. The early decrease in brachial artery diameter appears associated with the pressure drop (20 to 24 mmHg) that accompanies maximal brachial artery blood flow [7, 18]. The mechanisms responsible for this early vasoconstriction have not yet been fully explained but its existence was noted by Dobrosielski et al. [5] and recently confirmed to have a prevalence of above 60% in children and young adults [8, 19]. Because of our experimental design and the lack of a stereotactic stabilizer, we are unable to identify individuals with a true vasoconstrictor response since we lack an appropriate reference diameter.

We let our algorithm find the point of maximum diameter and the time to maximal diameter rather than perform a measurement at 60 seconds [3] based on observations [1, 4, 20] that the peak response does not necessarily occur at 60 seconds and found, on average, a peak-response at 55.2 seconds following cuff release. Variations in the time-to-peak dilation have been speculated to be secondary to factors linked to aging and level of physical fitness [21]. Our algorithm did not find a minimum diameter before a maximum in 765 members of our cohort (13.5% men and 14.3% women) (Fig 2D). We believe that there are two possibilities for this finding: (1) un-interpretable brachial artery diameter curves due to physiological vasoconstriction of the brachial artery or, (2) diameter mis-registration due to artifacts and noise. The first possibility was brought up by a group of investigators who described a negative flow mediated responses or brachial artery constriction (BAC) [22]. Sedlak et al found that 11% of their study population exclusively composed of women had such a response [22]. The second possibility is also likely given the known technical difficulties in obtaining precise brachial artery diameter measurements. We took into consideration both possibilities by assigning a TBAR value of 0.0% to these cases with the following logic: (1) if the response represented vasoconstriction, we conservatively biased the measurement to the null and, (2) if the error due to noise overwhelmed the TBAR, then the value of TBAR was likely low and close to zero.

Flow mediated dilation measured with respect to a diameter obtained before cuff inflation has been shown to be associated with CHD events [2, 17, 23, 24]. We add to these observations by reporting associations between CHD events and total brachial artery reactivity, a measurement made solely following the time when the occlusion cuff is deflated. Brachial artery reactivity calculated using a baseline diameter measured after cuff deflation might therefore be equivalent to the response seen with a baseline diameter measurement made before cuff inflation [25]. It has the advantage of limiting the errors associated with ultrasound probe displacement when a stereotactic device is not available.

Our study is limited in the following ways. It has technical limitations, may possess a participant selection bias, and is a multi-center study. We believe that the major limitations of our study are technical, being related to the acquisition protocol and the equipment used at the time of data analysis. All acquisitions were performed without the help of a stereotactic holder to help stabilize the position of the ultrasound transducer during an acquisition lasting up to 7 minutes [26]. This can cause sonographer fatigue and may therefore be a source of variability. The automated edge detection process used to determine brachial artery diameters took more than 10 minutes to generate the brachial diameter curves given the limitations of the computer hardware available in early 2000 (Pentium III processor; Intel Corporation, Santa Clara, CA). The length of the measurement process limited the reader’s ability to make adjustments to the region-of-interest position once processing was started. The process used to generate brachial artery diameter curves was therefore an automatic one with minimal operator involvement: one region-of-interest was drawn and the edge detector parameters were adjusted once.

At the time this study was undertaken there was no validated software that would have permitted the tracking of carotid and brachial artery diameters. The diameter tracking software was designed in house. Performance of this software was since validated in prior studies linking carotid diameters to left ventricular mass [27] and incident stroke [28]. Carotid artery distensibility measurements made with this software have also shown associations with left ventricular dynamics [29] and aortic wall calcification [30]. However, we also compared the ability of our software to perform traditional FMD measurements by comparing the results of measurements made with it to those obtained with Brachial Analyzer and the MESA software in a subset of 90 participants. Similar to the results of Faita et al., we found strong correlations between diameter measurements [31]. We found much weaker associations between techniques for FMD estimates. The strongest correlation was between our software and that used in MESA (Table 9).

Our TBAR reproducibility studies were performed in a completely blinded fashion. Due to time constraints and participant burden, the Coordinating Center at the University of Washington (Seattle, WA, USA) generated a list of participants that would have replicate studies. This was mostly done near the end of the study. The Ultrasound Reading Center received these studies with the subject identification numbers having been scrambled. Results of the analyses were matched after the analyses had been completed. Because of the design, almost all studies were performed by the same sonographer and read by the same reader. The timing of the replicate study is not known nor is any interval ingestion of food. As such, part of the variability might be secondary to these factors that are well recognized as modifying flow mediated dilation [32]. This might have contributed to the low correlation between replicate acquisitions.

The lack of cardiac gating might also have blunted associations between TBAR and events. However, diameter measurements made on images gated to diastole, to systole, or averaged throughout the cardiac cycle seem to give similar estimates of flow mediated dilation [33].

We address possible selection bias by listing the source of exclusions in Fig 1. This is either unexplained since the participant was not seen at the testing station, caused by a specific medical condition that precludes performance of the test, linked to the lack of availability of a digital record of the study at the time of processing, and an incomplete set of risk factors.

Given the range of responses reaching up to 190%, we performed one analysis where we excluded plausible outliers from our analyses (Fig 4). The results remained similar to those of the original analyses. We also looked at the scenario where we excluded cases with poorly defined maxima and minima (Fig 5). Comparing the Kaplan-Meier curves shown in Fig 4 to those on Fig 5, an incremental increase in the likelihood of events between quartiles 1 and 2 was subjectively more apparent. This might indicate that some of the responses that were set at 0.0% represented cases where the technical quality of the brachial artery diameter acquisitions was compromised.

One of the major limitations of our paper remains the lack of information on the magnitude of the nitric oxide (NO) release. While most studies in the literature do not capture this information, concurrent recording of the blood flow velocities (shear rate and shear stress) [34] as a surrogate of NO release might improve overall reliability of brachial artery flow mediated dilation and plausibly that of TBAR. However, even NO release only partly accounts for the flow mediated response of the brachial artery following release of an occlusion cuff [35, 36].

Local brachial artery distensibility might also be modulating the brachial artery responses during both the early decrease in diameter and the flow mediated increase in diameter. Witte et al. showed that decreased distensibility of the brachial artery is associated with decreased FMD responses [37]. It is not clear that brachial artery reactivity is a completely different phenotype than distensibility or is partly linked to it. This has implications for possible linkages between aging and heritability as modulators of local brachial artery compliance, large artery stiffening, and flow mediated dilation [38, 39]. For example, if brachial artery distensibility is linked to flow mediated dilation and distensibility is in part heritable, then flow mediated dilation may also have a genetic component. However, our experimental design does not permit us to address these issues directly. In a review of the factors associated with vascular aging, Paneni et al. point out a very likely linkage between endothelial dysfunction due to age related decreased nitric oxide production and increased breakdown [40]. A plausible effect of decreased NO activity on arterial smooth muscle tone would be to decrease distensibility. It is likely that this would also apply to FMD. Paneni et al. also suggest that certain negative traits may be passed on through epigenetic mechanisms and contribute to a milieu favoring vascular aging [40]. This might apply to the distensibility of muscular arteries and to flow mediated dilation.

Our comparisons between TBAR and FMD should be viewed from a qualitative perspective. The results, although showing statistical equivalence between both variables, should be viewed very cautiously. The MESA “classic” FMD measurements were made using a case-control design and, as such, a weighing scheme is needed to properly interpret the results. Using the available data to compare TBAR to FMD without a new weighing strategy limits the interpretation of the results.

The multi-center nature of our study implicitly introduced variability into the measurement process linked to the clinic site, number of sonographers, as well as limiting quality assurance processes due to off-site supervision by a core laboratory. A major limitation of our study includes imaging at six separate centers and by 20 different sonographers albeit 15 performed more than 20 studies each. The number of sonographers likely increased variability and attenuated the precision of our measurements. Such limitations can be overcome in specialized laboratories that study the brachial artery responses to reactive hyperemia in a systematic fashion [1, 25, 41, 42]. It is unclear whether this level of expertise can be consistently promulgated to the clinic. For example, some of the six clinic sites in our study hired trained and certified vascular sonographers while others relied on individuals with less formal training. However, one advantage of using total brachial artery reactivity as a measurement is limiting the length of data acquisition to a short time period following release of the occlusion cuff. This likely reduced measurement variability. While we recognize that our measurement process has greater variability than those generated in a specialized laboratory, our measurements of total brachial artery reactivity made solely in the time period when the occlusion cuff is released were significantly associated with coronary heart disease events.

### Conclusions

Total brachial artery reactivity is a significant independent predictor of first time coronary heart disease events. It is exclusively measured during the post-release phase of brachial reactivity studies. Further studies are needed to confirm this finding.
